# Perceived occupational HIV risk, stigma, and discriminatory attitudes among healthcare professionals in Jeddah and Riyadh, Saudi Arabia: a comparative cross-sectional study

**DOI:** 10.3389/fpubh.2026.1952225

**Published:** 2026-10-08

**Authors:** Amal I. Khalil, Seham M. Elgamal, Sitah S. Alshutwi, Waad Alsulami, Hanaa M. Aboshereda

**Affiliations:** 1 King Abdullah International Medical Research Center, Jeddah, Saudi Arabia; 2 College of Nursing, King Saud bin Abdulaziz University for Health Sciences, Jeddah, Saudi Arabia; 3 Ministry of National Guard Health Affairs (MNGHA), Jeddah, Saudi Arabia; 4 Faculty of Nursing, Menoufia University, Shebeen El-Kom, Egypt; 5 College of Nursing, King Saud bin Abdulaziz University for Health Sciences, Riyadh, Saudi Arabia; 6 College of Applied Medical Sciences, King Saud bin Abdulaziz University for Health Sciences, Jeddah, Saudi Arabia

**Keywords:** discriminatory attitudes, healthcare professionals, HIV, infection-control practices, institutional policies, perceived occupational risk, Saudi Arabia, stigma

## Abstract

**Background:**

Even with the presence of institutional policies, training programs, and various interventions, stigma associated with Human Immunodeficiency Virus (HIV) still obstructs fair access to healthcare services. In Saudi Arabia, there is a scarcity of research focusing on the regional variations among healthcare workers regarding their Fear of HIV Infection/Perceived Occupational Risk, their attitudes towards individuals living with HIV (PLHIV), and the stigma and discrimination they observe.

**Aim:**

This research aimed to compare these aspects among healthcare professionals in Riyadh and Jeddah.

**Methods:**

This study employed a comparative cross-sectional design involving 466 healthcare workers, including doctors, nurses, and allied health professionals, from selected healthcare facilities in Riyadh and Jeddah. Data collection was carried out using a validated, self-administered questionnaire that evaluated Fear of HIV Infection/Perceived Occupational Risk, attitudes towards PLHIV, observed stigma and discrimination, infection-control practices, the healthcare facility environment, and institutional policies. Descriptive statistics, comparisons between groups, and multiple regression analyses were utilized to explore regional differences and factors influencing attitudes towards PLHIV.

**Results:**

In Riyadh, participants tended to be older and possess more experience than those in Jeddah. Specifically, 39.7% of individuals from Riyadh were between 30 and 39 years old, while 55.0% of those from Jeddah were aged 20 to 29. Additionally, those in Riyadh were more likely to have extensive professional experience and prior training related to HIV. Both groups showed moderate levels of fear regarding HIV infection and perceived occupational risk, along with similar levels of support from institutional policies. Differences between regions were noted in terms of observed stigma and discrimination, with Riyadh scoring higher, and in infection-control practices, where Jeddah had slightly better scores. Multivariable regression analysis revealed minimal evidence that factors such as region, previous HIV-related training, fear of HIV infection, perceived occupational risk, or health facility policies independently influenced attitudes toward people living with HIV (R^2^ = 0.006, *p* = 0.596).

**Conclusion:**

In both areas, healthcare workers exhibited moderate levels of fear regarding HIV infection and perceived occupational risks. However, misconceptions and discriminatory attitudes towards people living with HIV (PLHIV) were still apparent. These results highlight the necessity of ongoing, evidence-based HIV education, initiatives to reduce stigma, and enhanced institutional support systems to foster compassionate and fair treatment of individuals with HIV in Saudi Arabia.

## Background

Human immunodeficiency virus (HIV) continues to pose a significant challenge to global public health, impacting medical, psychological, and social domains (1, 2). Although antiretroviral therapy (ART) has turned HIV into a condition that can be managed chronically, stigma and discrimination related to HIV persist in healthcare environments, hindering testing, treatment participation, adherence, and the overall quality of care (1, 2). Individuals living with HIV (PLHIV) might encounter negative perceptions, avoidance, breaches of confidentiality, or discriminatory actions that negatively influence their healthcare experiences and overall well-being (2).

While the prevalence of HIV in Saudi Arabia remains relatively low, the infection still demands ongoing public health focus. The country has been monitoring HIV since 1984, the year the first case was detected (3). According to recent estimates from UNAIDS, around 11,000 individuals were living with HIV in Saudi Arabia in 2023, with the adult prevalence rate for those aged 15–49 staying under 0.1%. Approximately 1,500 new HIV cases were estimated for that year (3, 4). These statistics highlight the ongoing need for early diagnosis, accessible treatment, prevention efforts, and healthcare free from stigma.

Stigma related to HIV among healthcare workers is complex and can include negative perceptions of people living with HIV (PLHIV), fear or perceived risk of contracting HIV at work, excessive infection-control measures, observed discriminatory actions, and organizational issues within healthcare settings (5, 6). Even with proper infection-control protocols in place, concerns about occupational exposure may continue, emphasizing the need to differentiate between fear of HIV infection and perceived occupational risk. Risk from factual HIV knowledge.

Earlier studies have indicated that factors such as professional training, prior interactions with PLHIV, workplace features, and institutional guidelines might influence healthcare workers’ attitudes and behaviors concerning HIV (7–9). Nonetheless, these associations are influenced by context, and cross-sectional studies cannot confirm if these elements directly affect stigma levels. Research in Saudi Arabia has faced limitations due to variations in sample sizes, professional diversity, geographic scope, and the stigma dimensions evaluated (8–12).

There is still a scarcity of evidence comparing various aspects of HIV-related stigma among healthcare professionals from different Saudi healthcare environments. Analyzing trends in Jeddah and Riyadh could pinpoint areas needing more research, while acknowledging that sample differences might also mirror their demographic and professional makeup. Station rather than geographic location alone. Therefore, this study aimed to compare Fear of HIV Infection/Perceived Occupational Risk, attitudes toward PLHIV, infection-control practices, observed stigma and discrimination, health facility environment, and health facility policies among healthcare professionals recruited from Jeddah and Riyadh, and to examine factors associated with HIV-related attitudes (4).

## Significance of the study

This research is vital as it addresses key gaps in understanding HIV-related stigma among healthcare professionals in Saudi Arabia, where cultural, religious, and legal factors uniquely influence attitudes and behaviors. By exploring these elements, the study helps develop interventions that are context-specific and culturally sensitive, aiming to reduce stigma and foster compassionate care. The findings are expected to guide policy development and offer evidence-based recommendations that nurture supportive healthcare environments for both providers and individuals living with HIV. Additionally, the study underscores the importance of education and training in enhancing providers’ knowledge, empathy, and cultural competence, thereby leading to improved patient-provider relationships and better health outcomes. Beyond Saudi Arabia, this research holds global relevance, enriching the understanding of HIV stigma across diverse cultures and advancing international efforts toward a stigma-free healthcare system.

## Theoretical framework

This research drew selectively on specific constructs from the Health Belief Model (HBM) and the Theory of Planned Behavior (TPB) to inform the conceptual organization and interpretation of the study variables; however, the study was not designed to fully operationalize or empirically test either theoretical model (13–15). As illustrated in Figure 1, selected constructs from these theories were conceptually aligned with the variables examined in the present study.

**Figure 1:**
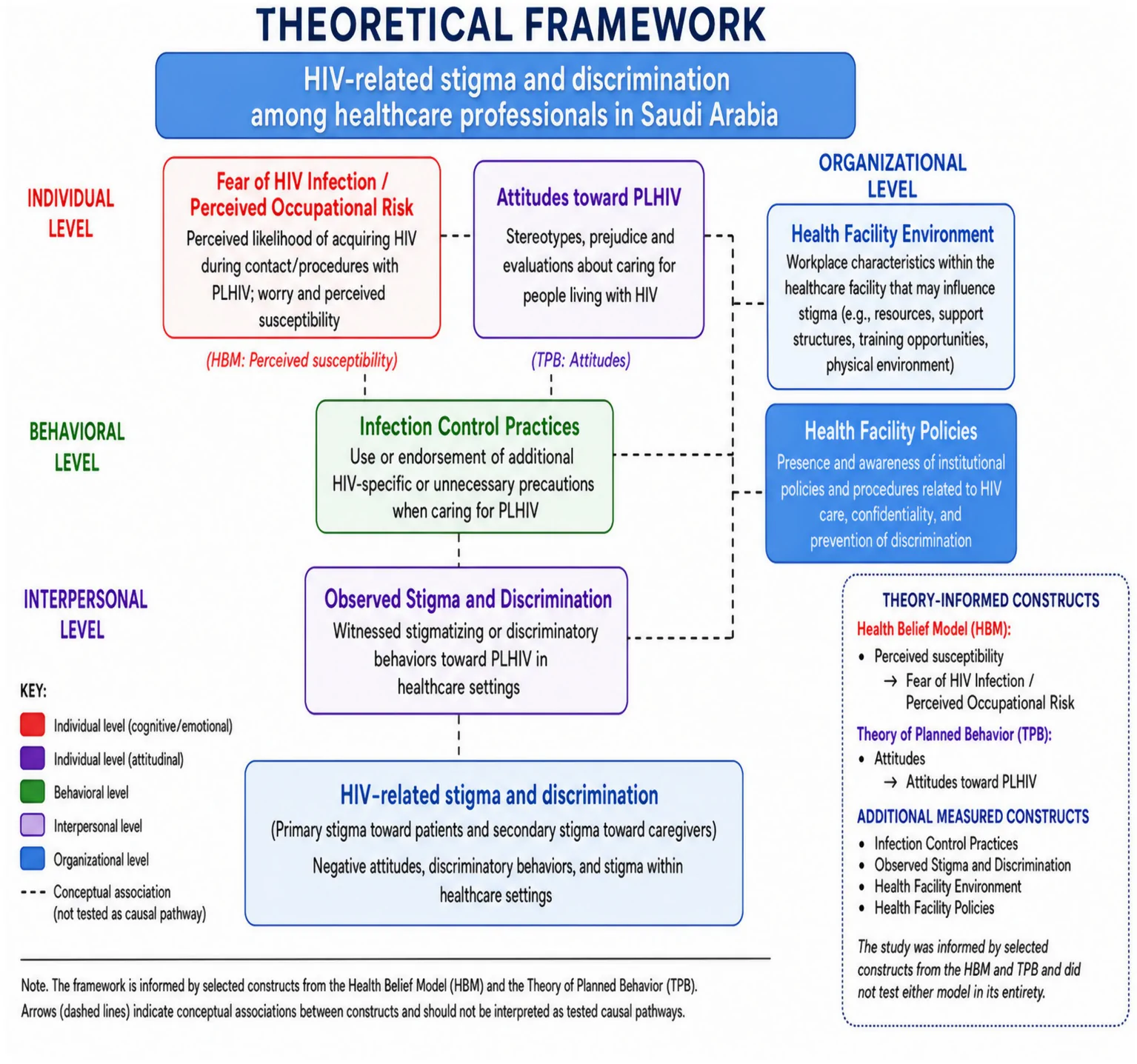
Theory-informed framework of HIV-related stigma and discrimination among healthcare professionals in Saudi Arabia. Selected constructs from the Health Belief Model and Theory of Planned Behavior were mapped to variables directly measured in the study. Perceived susceptibility was operationalized as Fear of HIV Infection/Perceived Occupational Risk, and TPB attitudes were operationalized as Attitudes toward PLHIV. Infection Control Practices, Observed Stigma and Discrimination, Health Facility Environment, and Health Facility Policies were treated as additional behavioral, interpersonal, and organizational constructs. Arrows represent conceptual relationships and should not be interpreted as tested causal pathways.

In relation to the HBM, perceived susceptibility was conceptually represented by Fear of HIV Infection/Perceived Occupational Risk, reflecting the extent to which healthcare professionals perceived themselves to be at risk of HIV infection while providing care for people living with HIV (PLHIV). Perceived susceptibility is a central component of the HBM and refers to an individual’s perception of vulnerability to a particular health threat (13, 14). Other core HBM constructs, such as perceived severity, perceived benefits, perceived barriers, cues to action, and self-efficacy, were not directly assessed by the study instrument and therefore were not included as measured theoretical variables.

With respect to the TPB, Attitudes Toward PLHIV were conceptually aligned with the attitudinal component of the theory, reflecting participants’ evaluations, opinions, and judgments regarding PLHIV. Within the TPB, attitudes toward a behavior, subjective norms, and perceived behavioral control contribute to behavioral intentions and subsequent behavior (15). In the present study, subjective norms, behavioral intentions, and perceived behavioral control were not directly measured. Although Infection Control Practices represent behavior-related responses within the clinical setting, they do not constitute a direct measure of perceived behavioral control as defined by the TPB. Therefore, Infection Control Practices were treated as a measured practice/behavioral outcome rather than as a TPB construct.

Observed Stigma and Discrimination, Health Facility Environment, and Health Facility Policies were incorporated as contextual variables rather than being assigned directly to either the HBM or TPB. This approach recognizes that HIV-related stigma and discriminatory practices operate across multiple levels, including individual, interpersonal, organizational, and broader structural contexts, and that healthcare environments and institutional policies can influence stigmatizing attitudes and practices (12). Accordingly, these domains were included to provide a broader contextual interpretation of HIV-related attitudes and practices within healthcare settings.

Overall, the framework was used to organize the measured variables across individual, attitudinal/behavioral, interpersonal, and organizational dimensions rather than to test the complete causal pathways proposed by either the HBM or TPB. Relationships among the measured variables were initially examined using bivariate analyses, while multivariable regression analysis was used to identify variables independently associated with attitudes toward PLHIV after adjustment for relevant demographic and professional characteristics. Thus, the application of the HBM and TPB in this study should be understood as theory-informed rather than as a definitive empirical test of either theoretical model.

### Aim of the study

The primary aim of this study was to evaluate HIV-related Fear of HIV Infection and discriminatory attitudes among healthcare professionals in Saudi Arabia and to identify factors associated with stigma.

### Specific objectives

1. Assess levels of HIV Fear of HIV Infection among physicians, nurses, and allied health staff.
2. Measure the prevalence and patterns of primary (patient-directed) and secondary (toward caregivers/staff) HIV-related stigma.
3. Compare Fear of HIV Infection and stigma across professions, regions, and clinical settings.
4. Identify demographic, professional, and psychosocial predictors of high stigma (e.g., years of experience, prior HIV training, Health Belief Model/Theory of Planned Behavior constructs).
5. Provide evidence-based recommendations for educational and institutional interventions to reduce stigma.

### Research questions

1. What is the level of HIV-related Fear of HIV Infection among healthcare providers?
2. What is the degree of discriminatory acts toward patients with HIV among healthcare providers?
3. What are the stigmatizing attitudes toward patients with HIV among healthcare providers?
4. What factors are associated with HIV-related Fear of HIV Infection, discriminatory behaviors, and stigmatizing attitudes among healthcare providers?
5. What are the relationships between socio-demographic characteristics and levels of HIV Fear of HIV Infection, discriminatory behaviors, and stigmatizing attitudes?
6. Are there differences in HIV-related Fear of HIV Infection between healthcare professionals in Riyadh and Jeddah?

### Hypotheses

H1: Higher HIV Fear of HIV Infection scores are associated with lower discriminatory attitudes.

H2: Professionals with prior HIV-specific training report significantly lower stigma than those without.

H3: Differences exist between professions, with nurses reporting higher perceived susceptibility and stigma compared with physicians (based on pilot data).

## Methods

### Study design

A comparative cross-sectional design was employed. This design is suitable for describing study variables at a fixed point in time and is widely used for exploratory and descriptive research (16, 17). Advantages include practicality, cost-effectiveness, and ease of implementation.

### Study setting

This study was conducted in three tertiary healthcare hospitals in Saudi Arabia located in Riyadh and Jeddah. These hospitals were selected because they serve large and diverse patient populations and employ substantial numbers of healthcare professionals from multiple disciplines. Following the receipt of all required institutional and ethical approvals in January 2024, data collection was conducted over an eleven-month period from February 2024 to December 2024. Data were collected by the principal investigator and co-investigators, who were responsible for participant recruitment and questionnaire administration across the participating hospitals. Standardized data collection procedures were implemented to ensure consistency throughout the study sites.

Study Population and Eligibility Participants were qualified healthcare professionals employed in the selected hospitals in Jeddah and Riyadh. The study population included physicians, nurses, nursing supervisors and matrons, social workers, paramedics, laboratory and radiology professionals, pharmacists and pharmacy technicians, and other allied healthcare professionals.

Healthcare professionals were eligible if they had completed their professional healthcare qualification, had been employed in the healthcare sector for at least 2 years following graduation, and were involved in patient care or the clinical supervision and oversight of patient-care delivery. Healthcare professionals who were concurrently undertaking postgraduate or further education remained eligible provided that they had already completed their professional qualification and met the required post-graduation professional experience criteria.

Inclusion Criteria • Qualified healthcare professionals employed in the selected hospitals for at least 2 years following graduation. • Involvement in patient care or clinical supervision and oversight of patient-care delivery. • Willingness to participate in the study.

Exclusion Criteria • Healthcare professionals with less than 2 years of post-graduation professional experience. • Undergraduate or pre-licensure students who had not yet qualified as healthcare professionals. • Personnel working exclusively in non-clinical administrative positions without involvement in patient-care delivery. • Individuals unwilling to participate.

### Sampling strategy and sample size determination

Sampling Strategy: A non-probability convenience sampling strategy was used to recruit eligible healthcare professionals from the participating healthcare institutions in Jeddah and Riyadh. Healthcare professionals who met the predefined eligibility criteria and were available during the data-collection period were invited to participate. Participation was entirely voluntary, and recruitment continued for more than 10 months to maximize participation across the selected institutions.

Because convenience sampling was used, recruitment depended on the accessibility and willingness of eligible healthcare professionals to participate (17). Equal recruitment from Jeddah and Riyadh was therefore not imposed as a regional quota. The final sample comprised 466 healthcare professionals, including 169 participants from Jeddah and 297 from Riyadh.

Accordingly, the sample reflects healthcare professionals who voluntarily participated from the selected institutions during the study period and should not be considered representative of all healthcare professionals in Jeddah, Riyadh, or Saudi Arabia. Although the extended recruitment period facilitated the inclusion of participants with diverse demographic and professional characteristics, the use of convenience sampling and voluntary participation limits the generalizability of the findings. The potential for selection bias associated with this non-probability sampling approach was therefore considered when interpreting the results.

Sample Size Determination Multiple approaches were used to estimate an adequate overall sample size for the planned statistical analyses. First, a statistical power calculation for multiple linear regression was conducted using the pwr package in R. Assuming a statistical power of 0.80, a medium effect size of f^2^ = 0.15, an alpha level of 0.05, and up to 40 predictors, the minimum required sample size was estimated at 273 participants.

Second, a population-based sample-size estimate was obtained using the Raosoft approach. Assuming an estimated target population of approximately 20,000 healthcare professionals, a 95% confidence level, a 5% margin of error, and a 50% response distribution, an estimated sample size of approximately 377 participants was obtained. This calculation was used as an additional conservative planning estimate for recruitment.

Sample size formulas used:


x=Z(c/100)2r(100−r).

n=(N−1)E2+xN·

E=n(N−1)(N−n)x.

where (N) represents the estimated population size, (r) represents the expected response distribution, (E) represents the acceptable margin of error, (c) represents the confidence level, (Z(c/100)) represents the critical value corresponding to the selected confidence level, and (n) represents the estimated required sample size. The final overall sample of 466 participants exceeded both the regression-based minimum requirement of 273 participants and the population-based planning estimate of approximately 377 participants. The unequal regional distribution of 169 participants from Jeddah and 297 from Riyadh reflects the convenience sampling approach and voluntary participation rather than failure to meet predetermined region-specific sample-size requirements.

### Operationalization of the theory-informed framework

Table 1 demonstrates the operational alignment between the theory-informed framework, the constructs measured in the present study, and the corresponding statistical analyses. Perceived susceptibility from the Health Belief Model was operationalized as Fear of HIV Infection/Perceived Occupational Risk, while the Theory of Planned Behavior attitude construct was operationalized as Attitudes toward PLHIV. Infection Control Practices, Observed Stigma and Discrimination, Health Facility Environment, and Health Facility Policies were treated as additional behavioral, interpersonal, and organizational constructs. Constructs not directly measured, including perceived severity, perceived benefits, subjective norms, and perceived behavioral control, were not empirically tested. Accordingly, the framework was used as a theory-informed structure rather than as a confirmatory test of the complete HBM or TPB.

**Table 1:** Operationalization of the theory-informed framework and corresponding analyses.

Theoretical/conceptual construct	Operationalization in this study	How examined
HBM: Perceived susceptibility	Fear of HIV Infection/Perceived Occupational Risk	Bivariate comparisons; included as a predictor in the regression model for Attitudes toward PLHIV
TPB: Attitudes	Attitudes toward PLHIV	Principal outcome in the multivariable linear regression model
Behavioral construct	Infection control practices	Bivariate comparisons across regional and participant characteristics
Interpersonal construct	Observed stigma and discrimination	Bivariate comparisons and associations with study characteristics
Organizational construct	Health facility environment	Bivariate comparisons and associations with study characteristics
Organizational construct	Health facility policies	Bivariate analyses; included as a predictor in the regression model for Attitudes toward PLHIV
HBM: Perceived severity	Not directly measured	Not tested
HBM: Perceived benefits	Not directly measured	Not tested
TPB: Subjective norms	Not directly measured	Not tested
TPB: Perceived behavioral control	Not directly measured	Not tested

HBM, Health Belief Model; TPB, Theory of Planned Behavior; PLHIV, people living with HIV. The study was informed by selected constructs from the HBM and TPB and was not designed as a confirmatory test of either model in its entirety.

### Data collection tools

Data were collected using a structured self-administered questionnaire developed on Google Forms. The survey link and QR code were distributed by the principal investigator and co-investigators to eligible healthcare professionals at the participating hospitals. Participants accessed and completed the questionnaire electronically after providing informed consent. That was designed to assess HIV-related stigma and discrimination of healthcare providers. The questionnaire consisted of two sections:

### Measurement instrument, scoring, validity, and reliability

#### Section 1: sociodemographic and professional characteristics

This section collected participants’ sociodemographic and professional characteristics, including age, sex, marital status, educational level, current professional role, years of professional experience, nature of work, previous HIV-related training, and prior experience providing care to people living with HIV (PLHIV).

#### Section 2: HIV stigma and discrimination measurement

HIV-related stigma and discrimination were assessed using items based on the *Measuring HIV Stigma and Discrimination Among Health Facility Staff* questionnaire developed by the Futures Group through the Health Policy Project (2013) (18). The instrument was developed through an international collaborative process and piloted in six countries: China, Dominica, Egypt, Kenya, Puerto Rico, and St. Kitts and Nevis (18, 19). In this study, we used the English version of the questionnaire. An Arabic version was not necessary because all participants were healthcare practitioners at the hospitals of the National Guard Health Affairs (NGHA) in Riyadh and Jeddah, where English is commonly spoken in professional communication, clinical records, education, and practice. The reliability of the questionnaire in the present sample was established as satisfactory, as the Cronbach’s alpha was found to be 0.70. The original instrument addresses four broad areas: fear of HIV infection among healthcare facility staff; stereotypes and prejudice toward PLHIV; observed and secondary stigma and discrimination; and policy and work-environment factors. The original five-item attitude scale demonstrated acceptable internal consistency, with a Cronbach’s alpha of 0.78 (19).

For the present study, relevant questionnaire items were organized into six analytic constructs according to the concept measured by the items: Fear of HIV Infection/Perceived Occupational Risk, Infection Control Practices, Attitudes toward PLHIV, Observed Stigma and Discrimination, Health Facility Environment, and Health Facility Policies. These represent the analytic constructs used in the present study rather than six independently validated subscales of the original questionnaire.

**Table:** 

Construct	No. of items	Scoring/range	Interpretation	Reliability in present study
Fear of HIV infection/perceived occupational risk	10	Items combined into a composite score according to their response coding	Higher scores indicate greater worry/perceived occupational susceptibility to HIV infection	Reliability coefficient = 0.70
infection control practices	2	1–5; mean of items	Higher scores indicate greater endorsement/use of additional HIV-specific precautions	Two-item reliability = 0.76
Attitudes toward PLHIV	8	1–5; mean after reverse coding where required	Higher scores indicate more negative/stigmatizing attitudes	Cronbach’s α = 0.81
Observed stigma and discrimination	3	1–5; mean of items	Higher scores indicate more frequently observed stigma/discrimination	Cronbach’s α = 0.73
Health facility environment	5	1–5; mean of items	Higher scores indicate a more supportive environment after consistent coding	Cronbach’s α = 0.88
Health facility policies	1	0 = No; 1 = Yes	A score of 1 indicates reported presence of the relevant institutional policy	Not applicable—single-item indicator

#### Fear of HIV infection/perceived occupational risk

The Fear of HIV Infection/Perceived Occupational Risk construct comprised 10 items. These items assessed participants’ worry or perceived susceptibility to HIV acquisition during contact with PLHIV or while undertaking healthcare-related procedures. The construct was previously labeled “Knowledge”; however, this terminology was revised because the items primarily assess perceived occupational risk and fear rather than factual HIV knowledge. Higher scores indicate greater fear of HIV infection or perceived occupational susceptibility.

Internal consistency of this 10-item construct was assessed in the present study and met the predefined criterion for acceptable reliability (coefficient = 0.70).

#### Infection control practices

The Infection Control Practices construct consisted of 2 items assessing the use or endorsement of additional HIV-specific infection-control precautions when caring for PLHIV. Responses were scored from 1 to 5, and the mean of the two items was used as the composite score. Higher scores indicate greater endorsement or use of additional HIV-specific precautions.

The two-item construct demonstrated acceptable reliability in the present sample (reliability coefficient = 0.76).

#### Attitudes toward PLHIV

The Attitudes toward PLHIV construct consisted of 8 items rated on a five-point Likert scale from 1 = strongly disagree to 5 = strongly agree. Positively worded items were reverse-coded before calculating the composite score to ensure that all items followed the same conceptual direction. Reverse coding followed the transformation 1 → 5, 2 → 4, 3 → 3, 4 → 2, and 5 → 1.

The mean of the eight items was calculated, producing a possible score ranging from 1 to 5, with higher scores indicating more negative or stigmatizing attitudes toward PLHIV.

The eight-item construct demonstrated acceptable internal consistency in the present sample (Cronbach’s α = 0.81). This current-study reliability should be distinguished from the previously published α = 0.78, which refers specifically to the original five-item attitude scale (19).

#### Observed stigma and discrimination

The Observed Stigma and Discrimination construct comprised 3 items assessing participants’ observations of stigmatizing or discriminatory behaviors toward PLHIV within healthcare settings. Responses were coded from 1 to 5, with higher values representing greater frequency of observed stigma or discrimination. The mean of the three items was calculated to obtain the composite score.

The construct demonstrated acceptable internal consistency in the present study (Cronbach’s α = 0.73).

#### Health facility environment

The Health Facility Environment construct consisted of 5 items assessing participants’ perceptions of institutional and interpersonal conditions surrounding HIV care, including workplace support and aspects of secondary stigma. Items were rated on a five-point scale from 1 = strongly disagree to 5 = strongly agree.

Where necessary, items were reverse-coded so that all responses followed a consistent conceptual direction before the composite mean was calculated. Higher scores indicate a more supportive health-facility environment. The five-item construct demonstrated acceptable internal consistency in the present study (Cronbach’s α = 0.88).

#### Health facility policies

The Health Facility Policies variable consisted of 1 binary item assessing the reported presence or awareness of the relevant institutional HIV-related policy or procedure. Responses were coded 0 = No and 1 = Yes.

Because this construct was represented by a single item, calculation of Cronbach’s alpha or another internal-consistency coefficient was not appropriate.

#### Handling of “not applicable” responses

When a “not applicable” response was available because a clinical procedure or activity did not fall within the participant’s professional responsibilities, the response was treated as non-scorable/missing for that item rather than being interpreted as either stigmatizing or non-stigmatizing. Composite means were calculated using available applicable responses.

### Validity and reliability

The original Health Policy Project instrument underwent multinational development, expert review, and field testing, supporting its content validity and applicability across healthcare settings (18, 19). The original five-item attitude scale demonstrated a Cronbach’s alpha of 0.78 (19).

For the present study, reliability was evaluated separately for the multi-item analytic constructs because they represent conceptually distinct dimensions. A reliability coefficient of 0.70 or greater was considered indicative of acceptable internal consistency. All constructs met this criterion, with reliability coefficients ranging from 0.70 to 0.88. All multi-item constructs met this criterion: Fear of HIV Infection/Perceived Occupational Risk (10 items), Infection Control Practices (2 items), Attitudes toward PLHIV (8 items), Observed Stigma and Discrimination (3 items), and Health Facility Environment (5 items). Internal-consistency reliability was not applicable to the single-item Health Facility Policies indicator.

### Ethical considerations for tool use

Permission to use the questionnaire and associated materials was obtained from the Futures Group, with proper citation, ensuring compliance with copyright and ethical standards.

## Data collection procedure

The study’s data were collected using a structured questionnaire that participants completed independently. To ensure consistent application across all research locations and adherence to ethical standards, the following measures were implemented:

1. Preparation and Coordination. Before data collection commenced, necessary approvals were secured from relevant institutional bodies, such as the Nursing Administration and Medical Education Departments at each participating healthcare facility. The research team collaborated with unit supervisors to schedule appropriate times for distributing the questionnaires, ensuring minimal disruption to clinical duties.
2. Training of Data Collectors. Research assistants responsible for distributing the questionnaires attended a brief orientation session. The training covered the study’s objectives, criteria for participant inclusion and exclusion, participant rights, confidentiality protocols, and standardized instructions for using the tool.
3. Participant Recruitment. Eligible participants, including doctors, nurses, and other healthcare workers, were approached in their respective departments.

The study was verbally explained using a standardized script, emphasizing its voluntary nature, anonymity, and the absence of risks or penalties for opting out. Participants who agreed to participate provided informed consent before receiving the questionnaire.

1. Questionnaire Distribution. The self-administered questionnaire was provided either in paper form or electronically via a secure survey link, depending on the site’s preference. Each questionnaire packet included an information sheet, a consent form, and clear instructions for completing the survey. Participants were given 10–15 min to complete the questionnaire in a private or semi-private setting to minimize peer influence.
2. Collection and Verification. Completed paper questionnaires were immediately collected in sealed envelopes to ensure confidentiality. Electronic responses were stored on a secure, password-protected server accessible only to the research team. The research team checked returned questionnaires for completeness, but participants were not pressured to answer any skipped questions to respect their autonomy.
3. Ensuring Anonymity and Confidentiality. No personal identifiers, such as names, IDs, or employee numbers, were collected. Each questionnaire was assigned a unique numerical code for data entry and analysis. All collected data were stored in encrypted files accessible only to the principal investigator.
4. Data Entry and Quality Control. Data were entered into a statistical software program (e.g., SPSS) by two independent researchers to minimize entry errors. A random sample (10%) of entries was cross-checked for accuracy. Any discrepancies were resolved through consensus with the research supervisor.

*Timeline*: Data collection was conducted over an 11-month period, from February 2024 to December 2024, to allow sufficient opportunity to recruit healthcare professionals working across different shifts, including morning, evening, and night duties. The extended data collection period also facilitated recruitment from different departments and work schedules, thereby allowing participation from healthcare professionals with varied working patterns and professional contexts.

## Data management and analysis plan

Collected data were coded, cleaned, tabulated, and analyzed using JMP Pro, version 19.1.3 (JMP Statistical Discovery LLC, Cary, NC, USA). Data completeness, coding accuracy, outliers, and distributional characteristics were examined before conducting the statistical analyses. Normality of continuous variables was assessed using the Shapiro–Wilk test, supported by visual inspection of histograms and Q–Q plots.

Continuous variables with approximately normal distributions were summarized as mean ± standard deviation (SD), whereas non-normally distributed continuous variables were summarized using the median and interquartile range (IQR). Categorical variables were reported as frequencies and percentages.

For comparisons between two independent groups, normally distributed continuous variables were analyzed using the independent-samples Student’s t test, whereas non-normally distributed variables were analyzed using the Mann–Whitney U test. For comparisons involving more than two independent groups, one-way analysis of variance (ANOVA) or the Kruskal–Walli’s test was used, as appropriate. Associations between categorical variables were examined using the chi-square test or Fisher’s exact test when expected cell frequencies were insufficient. Spearman’s rank correlation coefficient was used to assess associations between quantitative or ordinal variables when the assumptions for Pearson correlation were not met.

Comparisons of HIV-related study measures between Jeddah and Riyadh and across sociodemographic and professional characteristics were considered bivariate analyses. These analyses were used to characterize patterns within the study sample and were not interpreted as demonstrating independent or causal regional effects.

Multivariable linear regression analysis was used to examine factors associated with attitudes toward people living with HIV (PLHIV). Region was entered with Jeddah as the reference category, and HIV training was entered with No previous HIV training as the reference category. The construct previously labeled “Knowledge” was revised to Fear of HIV Infection/Perceived Occupational Risk to reflect the actual content of the items. Categorical predictors were entered using indicator/dummy coding with clearly defined reference categories.

Regression assumptions were evaluated before model interpretation. Linearity and homoscedasticity were examined using residual-versus-fitted plots, and the distribution of residuals was assessed using histograms and normal Q–Q plots. Multicollinearity was evaluated using tolerance statistics and variance inflation factors (VIFs), with VIF values <5 considered indicative of no problematic multicollinearity. Potentially influential observations were assessed using standardized residuals, leverage statistics, and Cook’s distance.

For statistically significant between-group comparisons, the magnitude of the difference was additionally evaluated using the absolute mean difference, 95% confidence interval (CI), and standardized effect size (Hedges’ g). Effect sizes were interpreted alongside statistical significance to distinguish statistical evidence from the potential substantive importance of the observed differences. Because established minimal clinically important differences are not available for these study-specific analytic constructs, statistically significant findings were not automatically interpreted as clinically or public-health meaningful.

Missing data were examined before analysis. Responses recorded as “not applicable” for clinical activities outside a participant’s professional role or experience were treated as non-scorable for the corresponding item rather than as either positive or negative responses. No imputation was performed, and analyses were conducted using available data for the relevant variables.

Regression results were reported as unstandardized regression coefficients (B), standard errors (SE), 95% confidence intervals (CIs), and two-sided *p* values. Greater emphasis was placed on the magnitude and precision of effect estimates and their 95% CIs rather than on statistical significance alone.

Given the large number of comparisons conducted across individual questionnaire items, HIV-related constructs, sociodemographic characteristics, and bivariate associations, these secondary analyses were regarded as exploratory and hypothesis-generating. No formal multiplicity adjustment was applied to these exploratory comparisons; therefore, their reported *p* values are nominal and should be interpreted cautiously, particularly when statistically significant findings occurred in isolation. Greater interpretive weight was given to the consistency of findings across analyses, effect estimates and confidence intervals, and multivariable results. All statistical tests were two-sided, and a nominal *p* value <0.05 was considered statistically significant.

## Results

Table 2 summarizes the sociodemographic and professional characteristics of the 466 participants, including 169 from Jeddah and 297 from Riyadh. Participants aged 20–29 years represented a larger proportion of the Jeddah sample than the Riyadh sample [93 (55.0%) vs. 123 (41.4%)], whereas those aged 30–39 years were more common in Riyadh than in Jeddah [118 (39.7%) vs. 47 (27.8%)]. Females comprised 136 (80.5%) participants in Jeddah and 212 (71.4%) in Riyadh, while Saudi nationals accounted for 122 (72.2%) and 149 (50.2%), respectively. Regarding professional role, nurses represented 43 (25.4%) participants in Jeddah compared with 9 (3.0%) in Riyadh, whereas other allied healthcare professionals represented 29 (17.2%) and 90 (30.3%), respectively. Nursing supervisors/matrons accounted for 6 (3.6%) participants in Jeddah and 14 (4.7%) in Riyadh. Previous HIV-related training was more frequently reported in Riyadh [190 (64.0%)] than in Jeddah [65 (38.5%)], while previous experience providing care to PLHIV was reported by 169 (56.9%) participants in Riyadh and 85 (50.3%) in Jeddah. The revised professional-role terminology clarifies that participants categorized as Nurse were qualified nursing graduates employed as healthcare professionals, including those undertaking further or postgraduate education, while Nursing supervisor/Matron referred to qualified nurses with clinical supervisory responsibilities. All participants met the prespecified eligibility criteria.

**Table 2:** Demographic and work-related characteristics of participants by City (Jeddah and Riyadh) *N* = 466.

Variables	Jeddah *n* (%)	Riyadh *n* (%)
Age		
<20 years*	1 (0.6)	0 (0.0)
20–29 years	93 (55.0)	123 (41.4)
30–39 years	47 (27.8)	118 (39.7)
40–49 years	19 (11.2)	42 (14.1)
≥50 years	9 (5.3)	14 (4.7)
Sex		
Female	136 (80.5)	212 (71.4)
Male	33 (19.5)	85 (28.6)
Nationality		
Saudi	122 (72.2)	149 (50.2)
Non-Saudi	47 (27.8)	148 (49.8)
Marital status		
Single	90 (53.3)	160 (53.9)
Married	72 (42.7)	135 (45.4)
Divorced	5 (3.0)	2 (0.7)
Widowed	2 (1.2)	0 (0.0)
Current professional role		
Nursing supervisor/Matron	**6 (3.6)**	**14 (4.7)**
Clinician	46 (27.2)	79 (26.6)
Other allied healthcare professional	29 (17.2)	90 (30.3)
Paramedic	16 (9.5)	47 (15.8)
Pharmacist	14 (8.3)	14 (4.7)
Physician	15 (8.9)	34 (11.5)
Nurse	**43 (25.4)**	**9 (3.0)**
Technician	0 (0.0)	0 (0.0)
Not classified/reported§	0 (0.0)	**10 (3.4)**
Education level		
Diploma	25 (14.8)	54 (18.2)
Bachelor’s degree	89 (52.7)	121 (40.8)
Master’s degree	35 (20.7)	67 (22.6)
PhD	20 (11.8)	55 (18.5)
Years in healthcare		
<5 years	Verify	121 (40.8)
5–10 years	40 (23.5)	70 (23.6)
>10 years	44 (25.9)	106 (35.7)
Department		
Administration (Nursing Matrons)	3 (1.8)	6 (2.0)
Ambulance	7 (4.1)	35 (11.8)
Emergency	26 (15.4)	89 (30.0)
ICU	13 (7.7)	25 (8.4)
Laboratory	8 (4.7)	9 (3.0)
Medicine	31 (18.3)	54 (18.2)
Nursing	14 (8.3)	43 (14.5)
Pharmacy	5 (3.0)	7 (2.4)
Psychiatry	2 (1.2)	0 (0.0)
Radiology	5 (3.0)	7 (2.4)
Surgery	23 (13.6)	37 (12.5)
Other	32 (18.9)	20 (6.7)
Nursing and Education	3 (1.8)	1 (0.3)
Oncology and Hematology	2 (1.2)	4 (1.3)
Pediatrics	1 (0.6)	2 (0.7)
Pharmacy and Laboratory Services	8 (4.7)	3 (1.0)
Rehabilitation and Therapy Services	3 (1.8)	1 (0.3)
Surgical Services	3 (1.8)	2 (0.7)
Previous HIV training		
Yes	65 (38.5)	190 (64.0)
No	104 (61.5)	107 (36.0)
Previously provided HIV care		
Yes	85 (50.3)	169 (56.9)
No	84 (49.7)	128 (43.1)

Percentages are column-wise (within Jeddah and within Riyadh). Values may not sum to 100% due to rounding. Bold text indicates the main demographic and work-related characteristic categories.

Table 3 shows the results of a Pearson chi-square test conducted to evaluate the association between sex and region. The findings showed a statistically significant association, χ^2^(1, N = 466) = 4.241, *p* = 0.039, suggesting that the distribution of sex differed notably between the two areas. The percentage of female participants was greater in Jeddah (80.5%) than in Riyadh (71.4%), whereas male participants were more prevalent in Riyadh (28.6%) than in Jeddah (19.5%). This result indicates a subtle yet significant gender disparity between the two groups, potentially highlighting regional variations in workforce makeup or hiring practices in healthcare organizations.

**Table 3:** Sex by region (counts and column percentages) and Pearson’s Chi-square *N* = 466.

Sex	Jeddah *n* (%)	Riyadh *n* (%)
Female	136 (80.5%)	212 (71.4%)
Male	33 (19.5%)	85 (28.6%)
Chi-square (χ^2^)	= 4.241, *p* = 0.039*	

Pearson’s chi-square test comparing sex distribution by region; *p < 0.05.

Table 4 displays the comparison of average scores on HIV-related subscales between participants from Jeddah and Riyadh, analyzed with the Mann–Whitney U test. The examination showed no statistically significant disparities between the two areas in attitudes (U = 24,329, *p* = 0.582), perceived occupational risk/fear of HIV infection (U = 25,310, *p* = 0.878), infection control (U = 27,044, *p* = 0.147), or health facility policies (U = 24,805, *p* = 0.831). These findings suggest equivalent levels of HIV-related attitudes, perceived occupational risk/fear of HIV infection, and infection control practices among healthcare providers in both areas, along with similar institutional policies concerning HIV management.

**Table 4:** Region (Jeddah vs. Riyadh) and continuous HIV discrimination scales (Mann–Whitney U) (*N* = 466).

Variable	Jeddah (Mean *±* SD)	Riyadh (Mean *±SD*)	U statistic	*p*-value
Attitudes	19.68 *±* 4.94	20.06 *±* 5.80	24,329	0.582
Fear of HIV infection/perceived occupational risk	8.78 *±* 3.86	8.84 *±* 3.80	25,310	0.878
Infection control	3.69 *±* 1.53	3.55 *±* 1.39	27,044	0.147
Observed stigma	8.64 *±* 2.75	9.24 *±* 2.74	21,645	0.013*
Health facility environment	10.99 *±* 5.16	12.32 *±* 6.10	21,985	0.025*
Health facility policies	11.59 *±* 1.60	11.61 *±* 1.52	24,805	0.831

Two-sided Mann–Whitney U tests comparing Jeddah vs. Riyadh for each scale. Values are Mean ± SD for context; inference is rank-based. *p < 0.05.

Nonetheless, notable regional variations were noted in observed stigma (U = 21,645, *p* = 0.013*) and health facility environment (U = 21,985, *p* = 0.025*). Individuals from Riyadh indicated higher observed stigma (9.24 ± 2.74) than those from Jeddah (8.64 ± 2.75), indicating that stigma might be more apparent or widespread in healthcare environments in Riyadh. In contrast, participants from Riyadh indicated a more favorable health facility environment (12.32 ± 6.10) than those in Jeddah (10.99 ± 5.16), likely highlighting variations in institutional preparedness, resource accessibility, or experiences with HIV-related training programs.

Figure 2 shows differences between Jeddah and Riyadh across HIV-discrimination subscales: Riyadh scores slightly higher on Attitudes (20.06 vs. 19.68), Fear of HIV Infection/ (8.84 vs. 8.78), Observed Stigma (9.24 vs. 8.64), and especially Health Facility Environment (12.32 vs. 10.99—the largest gap), while Health Facility Policies are virtually identical (11.61 vs. 11.59) and Jeddah is marginally higher on Infection Control (3.69 vs. 3.55).

**Figure 2:**
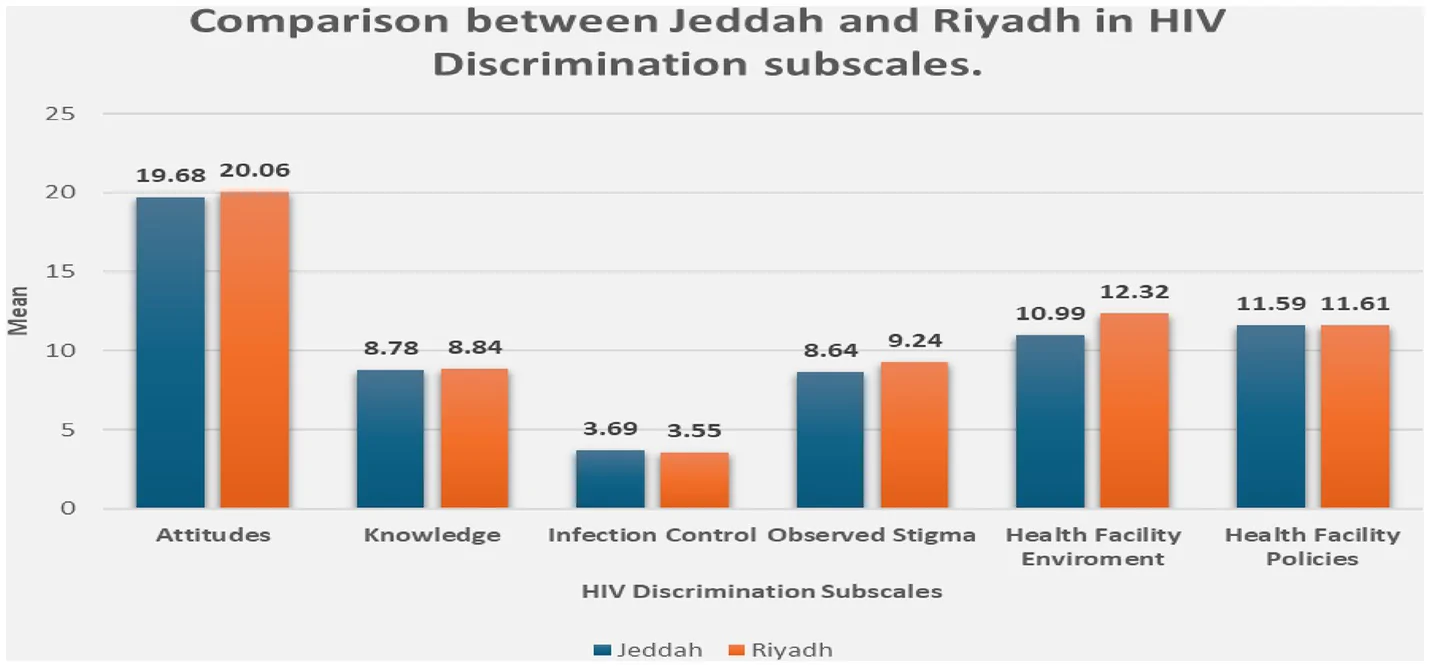
Comparison betweenJeddah and Rriyadh in relation to discrimatory attitudes scale.

Table 5 presents the Pearson correlation coefficients examining associations between participants’ HIV-related scales and facility-level measures. Attitudes toward HIV were not significantly associated with either the health facility environment (r = 0.074, *p* = 0.110) or health facility policies (r = 0.059, *p* = 0.201). A modest but significant positive correlation was found between Fear of HIV Infection and the health facility environment (r = 0.135, *p* = 0.003), suggesting that greater environmental support and resources were linked to higher levels of HIV related to Fear of HIV Infection. No significant relationships were observed between infection control and either facility measure.

**Table 5:** Pearson correlations of participant scales with facility measures.

X	Y	r	*p*-value
Attitudes	Health facility environment	0.074	0.110
Attitudes	Health facility policies	0.059	0.201
Fear of HIV infection/perceived occupational risk	Health facility environment	0.135*	0.003
Fear of HIV infection/perceived occupational risk	Health facility policies	−0.040	0.386
Infection control	Health facility environment	−0.012	0.794
Infection control	Health facility policies	0.046	0.320
Observed Stigma	Health facility environment	0.450*	< 0.0001
Observed Stigma	Health facility policies	−0.177*	< 0.001

Pearson’s r; two-sided tests. *p < 0.05. Extremely small *p*-values are thresholder for readability.

Observed stigma exhibited the strongest relationships among the variables, showing a significant positive association with the health facility environment (r = 0.450, *p* < 0.0001) and a significant negative association with health facility policies (r = −0.177, *p* < 0.001). These findings indicate that while a more supportive facility environment may coincide with more visible or more frequently reported expressions of stigma, stronger institutional policies were associated with lower levels of observed stigma, suggesting that policies may play a protective role independent of the physical or resource-based facility environment. Because of the cross-sectional design, this association does not establish the direction of the relationship or a causal effect of institutional policies on stigma. Overall, the results highlight the importance of both environmental and policy-level interventions in shaping fear of HIV infection/perceived occupational risk and reducing stigma among healthcare providers.

Table 6 presents the multivariable linear regression analysis examining factors associated with attitudes toward PLHIV. The overall regression model was not statistically significant, *F*(4, 461) = 0.694, *p* = 0.596, and explained only 0.6% of the variance in attitude scores (R^2^ = 0.006; adjusted R^2^ = −0.003). None of the included predictors demonstrated an independent statistically significant association with attitudes toward PLHIV. Compared with participants from Jeddah, those from Riyadh had an estimated 0.353-point higher attitude score; however, the estimate was imprecise and the 95% confidence interval included zero (B = 0.353, 95% CI − 0.721 to 1.426, *p* = 0.520). Similarly, previous HIV training (B = 0.105, 95% CI − 0.932 to 1.142, *p* = 0.843), Fear of HIV Infection/Perceived Occupational Risk (B = −0.052, 95% CI − 0.183 to 0.079, *p* = 0.436), and Health Facility Policies (B = 0.204, 95% CI − 0.120 to 0.528, *p* = 0.218) were not independently associated with attitudes toward PLHIV. Overall, the regression model provided little evidence that the included variables explained attitudes toward PLHIV, accounting for only 0.6% of the variance, with none of the individual predictors demonstrating a statistically significant independent association.

**Table 6:** Multivariable linear regression predicting attitudes toward PLHIV after adjustment for demographic and professional characteristics among healthcare professionals (*N* = 466).

Predictor	B	SE	95% CI Lower	95% CI Upper	*p*-value
Intercept	17.736	2.074	13.672	21.800	<0.001
HIV training: Yes, vs. No	0.105	0.529	−0.932	1.142	0.843
Region: Riyadh vs. Jeddah	0.353	0.548	−0.721	1.426	0.520
Fear of HIV infection/perceived Occupational risk	−0.052	0.067	−0.183	0.079	0.436
Health facility policies	0.204	0.165	−0.120	0.528	0.218

Model fit: N = 466; R^2^ = 0.006; adjusted R^2^ = −0.003; F(4, 461) = 0.694; p = 0.596; RMSE = 5.507.

Ordinary least-squares (OLS) regression was used. Reference categories were HIV training = No and Region = Jeddah. B values represent unstandardized regression coefficients. Two-sided p-values and 95% confidence intervals are reported. Higher scores on Fear of HIV Infection/Perceived Occupational Risk represent greater worry or perceived susceptibility to occupational HIV acquisition. *p* < 0.05 was considered statistically significant.

Figure 3 presents the unstandardized regression coefficient estimates and corresponding 95% confidence intervals for the predictors of attitudes toward people living with HIV (PLHIV), mirroring the results presented in Table 6. None of the selected predictors demonstrated a statistically significant independent association with attitudes toward PLHIV, as all 95% confidence intervals crossed zero. HIV training showed a small positive coefficient (B = 0.105, 95% CI − 0.932 to 1.142), indicating substantial uncertainty around the estimate. Participants recruited in Riyadh had a slightly higher estimated attitude score compared with those recruited in Jeddah (B = 0.353, 95% CI − 0.721 to 1.426); however, the wide confidence interval included zero, indicating that the regional association was not statistically significant. Fear of HIV Infection/Perceived Occupational Risk showed a small negative coefficient (B = −0.052, 95% CI − 0.183 to 0.079), while Health Facility Policies showed a small positive coefficient (B = 0.204, 95% CI − 0.120 to 0.528). Overall, the coefficient plot demonstrates that the estimated associations were small and imprecise, supporting cautious interpretation and indicating no clear independent association between the included predictors and attitudes toward PLHIV in this model.

**Figure 3:**
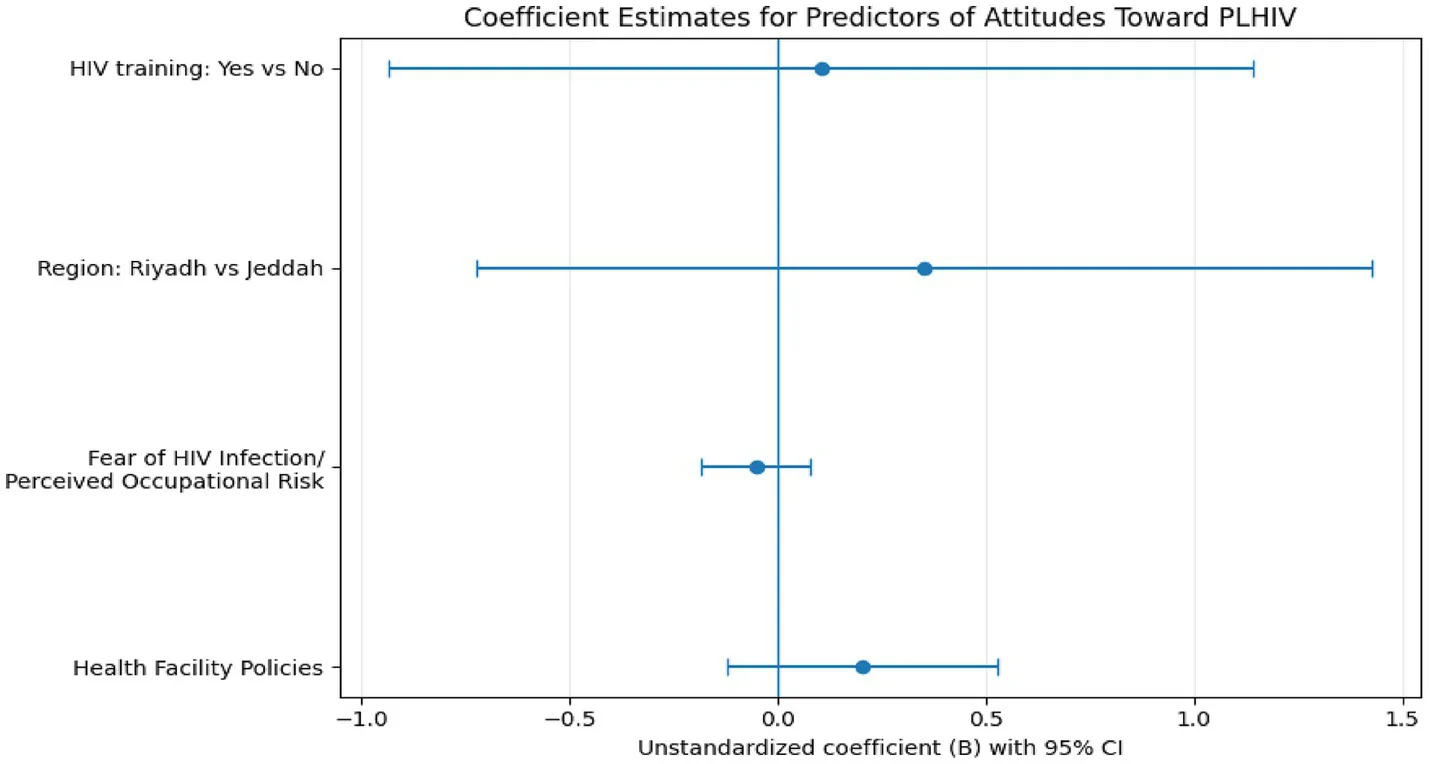
Coefficient plot of predictors of attitudes toward people living with HIV (PLHIV) among healthcare professionals. Points represent unstandardized regression coefficients (B), and horizontal lines represent 95% confidence intervals. The vertical reference line at B = 0 indicates no association. The model included HIV training, region, Fear of HIV Infection/Perceived Occupational Risk, and Health Facility Policies. Jeddah and no previous HIV training served as the respective reference categories.

Table 7 offers a comparative analysis of healthcare workers in Jeddah and Riyadh, focusing on three primary study areas: Fear of HIV Infection/Perceived Occupational Risk, infection control practices, and observed stigma toward people living with HIV (PLHIV). Within the Fear of HIV Infection/Perceived Occupational Risk domain, notable differences emerged between the two cities on several items. Participants from Riyadh expressed greater concern when performing both invasive and non-invasive procedures with PLHIV. Specifically, significant disparities were noted in tasks such as dressing wounds (χ^2^(4) = 25.42, *p* < 0.0001), drawing blood (χ^2^(4) = 9.615, *p* = 0.047), and taking the temperature of an HIV-positive patient (χ^2^(4) = 12.95, *p* = 0.011). In each instance, healthcare workers in Riyadh showed more apprehension compared with their counterparts in Jeddah. However, no significant difference was found regarding touching the clothing of a person living with HIV (χ^2^(4) = 3.57, *p* = 0.467). These results imply that healthcare workers in Riyadh perceive greater risk and experience greater anxiety related to HIV exposure, possibly indicating a lack of confidence in their infection prevention knowledge and practices.

**Table 7:** Comparison of HIV-related study measures between healthcare professionals in Jeddah and Riyadh (*N* = 466).

Item	Jeddah	Riyadh	$\chi_{d.f}$	*p*-value
Fear of HIV infection/perceived occupational risk				
*13.[Touched the clothing of a patient living with HIV]*				
Not applicable	38 (8.15)	86 (18.45)	3.574	0.467
Not worried	7 (1.5)	17 (3.65)		
A little worried	69 (14.81)	114 (24.46)		
Worried	15 (3.22)	21 (4.51)		
Very worried	40 (8.58)	59 (12.66)		
*13. [Dressed the wounds of a patient living with HIV]*				
Not applicable	29 (6.22)	85 (18.24)	25.424	<0.0001*
Not worried	17 (3.65)	15 (3.22)		
A little worried	24 (5.15)	47 (10.09)		
Worried	62 (13.3)	58 (12.45)		
Very worried	37 (7.94)	92 (19.74)		
*13. [Drew blood from a patient living with HIV]*				
Not applicable	21 (4.51)	59 (12.66)	9.6154	0.047*
Not worried	21 (4.51)	21 (4.51)		
A little worried	23 (4.94)	50 (10.73)		
Worried	65 (13.95)	92 (19.74)		
Very worried	39 (8.37)	75 (16.09)		
*13. [Took the temperature of a patient living with HIV]*				
Not applicable	42 (9.01)	71 (15.24)	12.954	0.011*
Not worried	20 (4.29)	16 (3.43)		
A little worried	71 (15.24)	108 (23.18)		
Worried	12 (2.58)	36 (7.73)		
Very worried	24 (5.15)	66 (14.16)		
Infection control				
*14. [Avoid physical contact]*				
Not Applicable	74 (15.88)	115 (24.68)	1.222	0.541
Yes	21 (4.51)	43 (9.23)		
No	74 (15.88)	139 (29.83)		
*14. [Wear double gloves]*				
Not Applicable	57 (12.23)	98 (21.03)	0.972	0.613
Yes	16 (3.43)	21 (4.51)		
No	96 (20.6)	178 (38.2)		
*14. [Use any special infection-control measures with patients living with HIV that you do not use with other patients]*				
Not Applicable	43 (9.23)	50 (10.73)	5.182	0.074
Yes	20 (4.29)	35 (7.51)		
No	106 (22.75)	212 (45.49)		
Observed stigma				
*15. [Healthcare workers unwilling to care for a patient living with or thought to be living with HIV]*				
Never	11 (2.36)	17 (3.65)	1.853	0.603
Once or twice	97 (20.82)	167 (35.84)		
Several times	51 (10.94)	85 (18.24)		
Most of the time	10 (2.15)	28 (6.01)		
*15. [Healthcare workers providing poorer quality of care to a patient living with or thought to be living with HIV, relative to other patients]*				
Never	10 (2.15)	13 (2.79)	11.623	0.008*
Once or twice	113 (24.25)	158 (33.91)		
Several times	26 (5.58)	60 (12.88)		
Most of the time	20 (4.29)	66 (14.16)		
*15. [Healthcare workers talking badly about people living with or thought to be living with HIV]*				
Never	9 (1.93)	17 (3.65)	7.523	0.056
Once or twice	113 (24.25)	161 (34.55)		
Several times	31 (6.65)	80 (17.17)		
Most of the time	16 (3.43)	39 (8.37)		
*16. How hesitant are healthcare workers in this facility to work alongside a coworker living with HIV, regardless of their duties?*				
Not hesitant	59 (12.66)	120 (25.75)	2.963	0.397
A little hesitant	58 (12.45)	85 (18.24)		
Somewhat hesitant	37 (7.94)	58 (12.45)		
Very hesitant	15 (3.22)	34 (7.3)		
*17. In the past 12 months, have you seen a person living with HIV in your health facility?*				
Yes	50 (10.73)	80 (17.17)	2.962	0.233
No	90 (19.31)	146 (31.33)		
Do not know	29 (6.22)	71 (15.24)		

* *p*-value is significant < 0.05.

Comparisons presented in this table are based on bivariate analyses between the two regional groups.

In the infection control domain, no statistically significant differences were found between Jeddah and Riyadh. The rates of physical contact avoidance (χ^2^(2) = 1.22, *p* = 0.541), use of double gloves (χ^2^(2) = 0.97, *p* = 0.613), and special infection-control measures not applied to other patients (χ^2^(2) = 5.18, *p* = 0.074) were similar in both regions. Nonetheless, the near-significant trend for using special precautions in Riyadh suggests a tendency toward excessive caution or fear rather than adherence to evidence-based infection control guidelines.

Regarding observed stigma, the results were mixed. A significant difference was found in perceptions of poorer quality of care for PLHIV (χ^2^ (3) = 11.62, *p* = 0.008), with Riyadh participants more frequently witnessing discriminatory practices (14.2% “most of the time” vs. 4.3% in Jeddah). Although not statistically significant, higher instances of negative talk about PLHIV (χ^2^ (3) = 7.52, *p* = 0.056) were also reported in Riyadh, suggesting a possible pattern of increased stigmatizing attitudes. No significant differences were observed regarding unwillingness to care for PLHIV (χ^2^ (3) = 1.85, *p* = 0.603) or hesitancy to work alongside HIV-positive coworkers (χ^2^ (3) = 2.96, *p* = 0.397). Lastly, exposure to PLHIV cases in the past year did not significantly differ between the two groups (χ^2^ (2) = 2.96, *p* = 0.233), although a higher percentage of respondents from Riyadh (17.2%) reported encountering PLHIV compared with those in Jeddah (10.7%).

Table 8 shows the comparison of mean scores (± standard deviation) for the six main HIV-discrimination scales across key demographic groups of healthcare workers (n = 466). First, there are consistent and statistically significant differences in Attitudes, Fear of HIV Infection/Perceived Occupational Risk, Observed Stigma, and perceptions of the Health Facility Environment across some demographic characteristics (*p* < 0.05). For example, Saudi vs. Non-Saudi, Muslim vs. Non-Muslim, and higher vs. lower education levels show meaningful differences in Attitudes toward people living with HIV and in Fear of HIV Infection/Perceived Occupational Risk scores, with Saudis, Muslims, and those with PhD-level education generally reporting higher Attitudes scores and Muslims reporting higher Fear of HIV Infection/Perceived Occupational Risk scores. Fear of HIV Infection/Perceived Occupational Risk also differs significantly by marital status and education level, with single participants and those with bachelor’s degrees tending to score higher than some other groups.

**Table 8:** Comparison of HIV-related study constructs across sociodemographic characteristics among healthcare professionals (*N* = 466).

Variables	Gender		Nationality		Religion		Region		Marital status				Education level			
	Male	Female	Saudi	Non-Saudi	Muslim	Non-muslim	Jeddah	Riyadh	Single	Married	Divorced	Widowed	Bachelor	Diploma	Master	PhD
	Mean ± Standard deviation															
	*P*-value															
Attitudes	20.05 ± 5.76	19.88 ± 5.42	20.52 ± 5.81	19.10 ± 4.94	20.34 ± 5.82	18.73 ± 4.27	19.68 ± 4.94	20.06 ± 5.80	20.82 ± 5.26	18.84 ± 5.53	19.78 ± 5.82	27.00 ± 7.07	19.69 ± 5.22	19.03 ± 6.51	21.10 ± 7.43	22.41 ± 5.37
	0.993		0.005*		0.006*		0.582		< 0.0001*				0.036*			
Fear of HIV infection/perceived occupational risk	8.66 ± 3.59	8.87 ± 3.89	9.20 ± 3.82	8.29 ± 3.76	9.15 ± 3.82	7.86 ± 3.67	8.78 ± 3.86	8.84 ± 3.80	9.66 ± 3.74	7.99 ± 3.77	7.61 ± 3.17	8.00 ± 5.66	9.17 ± 3.83	7.92 ± 3.78	6.35 ± 2.62	7.56 ± 3.47
	0.409		0.004*		< 0.0001*		0.878		< 0.0001				< 0.0001			
Infection Control	3.75 ± 1.23	3.55 ± 1.50	3.66 ± 1.51	3.52 ± 1.33	3.67 ± 1.44	3.40 ± 1.44	3.69 ± 1.53	3.55 ± 1.39	3.56 ± 1.51	3.67 ± 1.35	3.48 ± 1.50	3.00 ± 0.00	3.63 ± 1.49	3.57 ± 1.14	3.80 ± 1.20	3.31 ± 1.26
	0.316		0.413		0.173		0.147		0.591				0.416			
Observed Stigma	9.27 ± 2.95	8.94 ± 2.69	9.25 ± 2.76	8.70 ± 2.73	9.30 ± 2.83	8.21 ± 2.36	8.64 ± 2.75	9.24 ± 2.74	9.27 ± 2.73	8.72 ± 2.72	9.17 ± 3.16	8.50 ± 4.95	9.05 ± 2.67	9.70 ± 3.17	7.50 ± 3.33	8.87 ± 2.67
	0.327		0.019*		< 0.0001		0.013*		0.161				0.013*			
Health facility environment	12.45 ± 5.81	11.63 ± 5.80	12.11 ± 5.85	11.46 ± 5.74	12.25 ± 5.87	10.65 ± 5.46	10.99 ± 5.16	12.32 ± 6.10	12.02 ± 5.88	11.40 ± 5.74	13.87 ± 5.55	11.50 ± 0.71	11.57 ± 5.76	13.03 ± 7.07	12.95 ± 4.71	12.64 ± 5.31
	0.054		0.031*		< 0.0001		0.025*		0.050				0.040*			
Health facility policies	11.52 ± 1.54	11.63 ± 1.55	11.51 ± 1.55	11.72 ± 1.53	11.56 ± 1.62	11.70 ± 1.31	11.59 ± 1.60	11.61 ± 1.52	11.48 ± 1.54	11.72 ± 1.58	11.70 ± 1.26	12.50 ± 0.71	11.59 ± 1.54	11.32 ± 1.58	11.85 ± 1.69	11.82 ± 1.48
	0.425		0.137		0.614		0.831		0.247				0.356			

* *p*-value is significant < 0.05.

The comparisons presented are bivariate analyses. Potential confounding was further examined using multivariable regression analyses.

Observed Stigma and Discrimination scores differed significantly across several demographic characteristics, including religion, region, and educational level, while Health Facility Environment scores differed by nationality, religion, region, marital status, and educational level. In contrast, Infection Control Practices and Health Facility Policies showed no statistically significant differences across most demographic characteristics (all *p* > 0.05). For the regional comparisons, however, the magnitude of the statistically significant differences was small: Observed Stigma and Discrimination was 0.60 points higher in the Riyadh sample than in the Jeddah sample (95% CI 0.08–1.12; Hedges’ g = 0.22), while Health Facility Environment differed by 1.33 points (95% CI 0.28–2.38; Hedges’ g = 0.23). Both standardized effect sizes were small, indicating that these statistically significant regional differences should not be interpreted as necessarily reflecting substantial practical or public-health importance. Given the exploratory nature of the multiple bivariate comparisons, these findings should be interpreted cautiously and should not be attributed to differences in preparedness, resources, training, or institutional capacity because these potential explanatory mechanisms were not directly measured.

Table 9 displays the correlation coefficients and *p*-values for the six HIV-discrimination subscales: Attitudes, Fear of HIV Infection/Perceived Occupational Risk, Infection Control, Observed Stigma, Health Facility Environment, and Health Facility Policies among healthcare professionals. Multiple statistically significant relationships were detected. Notably, Observed Stigma demonstrated a moderate positive relationship with the Health Facility Environment (r = 0.45, *p* < 0.0001). In contrast, Observed Stigma showed a negative correlation with Health Facility Policies (r = −0.18, *p* = 0.0001), suggesting that more robust institutional policies could be associated with reduced observed stigma. Moreover, minor yet important positive correlations were observed between Fear of HIV Infection/Perceived Occupational Risk and both Observed Stigma (r = 0.11, *p* = 0.017) and Health Facility Environment (r = 0.14, *p* = 0.003), suggesting that greater fear of occupational HIV exposure was modestly associated with higher levels of observed stigma and more favorable perceptions of the health facility environment. No notable correlations were observed between Infection Control and the other subscales.

**Table 9:** Correlation matrix between HIV discrimination subscales among healthcare workers (*n* = 466).

Subscales	Attitudes	Perceived occupational risk/fear of HIV infection	Infection control	Observed stigma	Health facility environment	Health facility policies
	Correlation coefficient					
	*P*-value					
Attitudes	1	−0.038	−0.055	0.103	0.074	0.059
	0	0.411	0.232	0.026*	0.110	0.201
Fear of HIV infection/perceived occupational risk	−0.038	1	0.067	0.111	0.135	−0.04
	0.411	0	0.147	0.017*	0.003*	0.386
Infection control	−0.055	0.067	1	−0.072	−0.012	0.046
	0.232	0.147	0	0.123	0.794	0.320
Observed stigma	0.103	0.111	−0.072	1	0.45	−0.177
	0.026*	0.017	0.123	0	< 0.0001*	0.0001
Health facility environment	0.074	0.135	−0.012	0.45	1	−0.135
	0.110	0.003*	0.794	< 0.0001*	0	0.004*
Health facility policies	0.059	−0.04	0.046	−0.177	−0.135	1
	0.201	0.386	0.320	0.0001*	0.004*	0

* *p*-value is significant < 0.05.

## Discussion

This study examined HIV-related stigma and discrimination among healthcare professionals recruited from Jeddah and Riyadh, focusing on Fear of HIV Infection/Perceived Occupational Risk, attitudes toward PLHIV, infection-control practices, observed stigma and discrimination, health facility environment, and health facility policies. Overall, the findings did not demonstrate broad differences across all HIV-related constructs between the two samples. Attitudes toward PLHIV, Fear of HIV Infection/Perceived Occupational Risk, Infection Control Practices, and Health Facility Policies were broadly comparable between participants recruited from Jeddah and Riyadh. Differences were observed only for Observed Stigma and Discrimination, as well as Health Facility Environment, and these differences were small in magnitude.

### Workforce composition and between-group differences

The Jeddah and Riyadh samples differed substantially in several demographic and professional characteristics, including age, years of healthcare experience, previous HIV-related training, and experience caring for PLHIV. Participants recruited from Riyadh were generally older and more experienced, and reported greater previous exposure to HIV care, whereas the Jeddah sample included a higher proportion of younger and early-career healthcare professionals. These characteristics describe the composition of the recruited samples and should not be interpreted as inherent differences between the two geographic regions.

Given these differences in sample composition, between-group comparisons require caution. Previous studies have shown that professional experience, previous contact with PLHIV, HIV-related training, and demographic characteristics may be associated with healthcare professionals’ attitudes and stigma-related responses (20, 21). Nevertheless, the present findings do not support a generalized conclusion that healthcare professionals in one region systematically experience or demonstrate greater HIV-related stigma than those in the other. Statistically significant differences were limited to selected dimensions of the study measures.

### Attitudes, perceived occupational risk, and infection-control practices

Attitudes toward PLHIV did not differ significantly between participants recruited from Jeddah and Riyadh. Similarly, Fear of HIV Infection/Perceived Occupational Risk, previously labeled “Knowledge,” did not differ significantly at the overall construct level. The revised terminology better reflects that the items assess worry or perceived susceptibility during contact with or clinical procedures involving PLHIV rather than factual HIV knowledge.

Several individual occupational-risk items showed differences between the two groups. However, these item-level findings were part of multiple exploratory comparisons and should not be interpreted as evidence of systematic differences in perceived risk. They may identify specific areas for further investigation but require confirmation in studies with prespecified hypotheses.

Infection Control Practices were also broadly comparable between the two groups. Therefore, the present results do not support attributing differences in stigma to deficiencies in infection-control practice or factual knowledge. Previous studies have reported that fear of occupational infection, misconceptions regarding transmission, and uncertainty about contact with PLHIV may coexist with stigmatizing attitudes among healthcare professionals (22–25). However, the present cross-sectional findings cannot determine whether these factors contribute causally to stigma.

### Observed stigma and health facility environment

Observed Stigma and Discrimination scores were higher among participants recruited from Riyadh than among those recruited from Jeddah. However, the absolute difference was only 0.60 points (95% CI 0.08–1.12; Hedges’ g = 0.22), representing a small standardized effect. Similarly, Health Facility Environment scores differed by 1.33 points (95% CI 0.28–2.38; Hedges’ g = 0.23), also indicating a small effect.

Thus, although these differences reached nominal statistical significance, their practical or public-health importance should not be inferred from *p*-values alone. No established minimal clinically important difference is available for these analytic constructs, and both standardized effects were small. Moreover, because the two samples differed substantially in demographic and professional composition, these findings should not be attributed solely to geographic region.

Previous research has suggested that organizational climate, institutional support, workplace policies, and the broader healthcare environment may be associated with HIV-related stigma among healthcare professionals (20, 26, 27). In the present study, however, differences in Health Facility Environment scores cannot be attributed specifically to preparedness, resource availability, workplace culture, training intensity, or institutional support because these mechanisms were not directly measured. Such explanations therefore remain hypotheses rather than findings.

### Sociodemographic and professional associations

Exploratory bivariate analyses identified associations between selected demographic characteristics and several HIV-related constructs. For example, nationality, religion, marital status, and educational level were associated with some attitude, perceived-risk, observed-stigma, and facility-environment scores. Previous studies have similarly reported variation in HIV-related attitudes according to demographic, cultural, educational, and professional characteristics (20, 22, 26).

Nevertheless, these findings should be regarded as hypothesis-generating. Given the number of comparisons performed, isolated statistically significant *p*-values may have occurred by chance. Furthermore, associations with demographic characteristics should not be interpreted as evidence that these characteristics themselves cause HIV-related stigma. Future studies specifically designed to examine these relationships are required before substantive conclusions can be drawn.

### Factors associated with attitudes toward PLHIV

The multivariable regression findings are particularly important for interpretation. The overall model predicting attitudes toward PLHIV was not statistically significant, *F* (4, 461) = 0.694, *p* = 0.596, and explained only 0.6% of the variance in attitudes (R^2^ = 0.006; adjusted R^2^ = −0.003). None of the included predictors—region, previous HIV training, Fear of HIV Infection/Perceived Occupational Risk, or Health Facility Policies—showed a statistically significant independent association with attitude scores.

Accordingly, the results provide little evidence that these variables explain meaningful variation in attitudes toward PLHIV in this sample. Previous literature has proposed that HIV-related attitudes among healthcare professionals may be influenced by a complex combination of fear of infection, moral beliefs, social stigma, cultural perceptions, previous clinical contact with PLHIV, and organizational factors (20, 22–26). However, because many of these factors were not directly assessed in the present study, they should be regarded as possible hypotheses for future research rather than explanations established by the current findings.

### Interpretation in the Saudi healthcare context

HIV-related stigma and discrimination are multidimensional phenomena that may involve individual perceptions, interpersonal interactions, professional practices, and organizational conditions (20, 26, 28, 29). In the present study, most measured constructs were similar between participants recruited from Jeddah and Riyadh, while the few statistically significant between-group differences were small in magnitude.

Consequently, the findings do not support strongly differentiated intervention recommendations for Riyadh and Jeddah based on the present data alone. Instead, they support continued attention to stigma reduction across healthcare settings. Previous research has suggested that stigma-reduction strategies may include evidence-based HIV education, reinforcement of standard infection-control practices, respectful communication, contact-based learning, and clear institutional non-discrimination policies (20, 26, 28, 30). However, future intervention studies are needed to determine whether these approaches produce meaningful reductions in stigma within Saudi healthcare settings.

### Multiple comparisons and interpretation of associations

The item-level and sociodemographic analyses were exploratory and involved multiple comparisons. Therefore, isolated statistically significant findings were interpreted cautiously, with greater emphasis placed on effect magnitude, confidence intervals where available, consistency across analyses, and the multivariable findings. Associations identified only in bivariate analyses should be considered hypothesis-generating and require confirmation in future studies using prespecified hypotheses and appropriate multiplicity-control strategies.

More broadly, all findings should be interpreted within the constraints of the cross-sectional design. The present study identifies associations and patterns but cannot establish temporal sequence, directionality, effectiveness, or causality. Explanations involving workplace culture, resources, training, institutional support, religion, or social norms are therefore discussed only as possible mechanisms suggested by previous literature (31–34) and not as findings demonstrated by the present data.

### Strengths and limitations

This research sheds light on HIV stigma and discrimination, based on the responses of 466 healthcare practitioners selected from two Saudi cities namely Jeddah and Riyadh. The advantages of the study are that the sample consists of a large number of participants from different locations and professions, which allows measuring various aspects of HIV stigma in healthcare settings.

Yet, there are several drawbacks to be taken into account when interpreting the data. To start with, the cross-sectional nature of the design discourages any conclusions regarding the order of events or causality. Also, since convenience sampling was applied, the findings cannot be generalized to the general population. In addition to that, the samples from Jeddah and Riyadh had a number of significant differences in terms of demographics and professional experience, which makes us believe that disparities in the study groups may be caused not only by the geographic factors but by sample composition.

Thirdly, the data was collected with the help of self-reported questionnaires and is likely to have been influenced by recall and social desirability as the topic is very sensitive. In addition to that, information about infection-control practices and discrimination was based on self-reporting rather than actual clinical practices.

Fourthly, without the multiplicity adjustment, numerous exploratory comparisons among the questionnaire items, sociodemographic variables, and study constructs were performed, which may contribute to the increased probability of a type I error. Therefore, findings that are considered statistically significant must be interpreted with caution.

Lastly, despite the international validation and development of the HIV stigma and discrimination instrument, the constructs for analysis utilized in this study were aimed at meeting study purposes. Further psychometric validation in populations from Saudi healthcare will strengthen the statistical reliability and construct validity of these analytic constructs.

## Conclusion

The healthcare personnel employed in Jeddah and Riyadh varied due to the differences in different demographic and professional characteristics like age, years of experience working in healthcare, previous HIV-related training as well as HIV care. The Riyadh sample included more experienced professionals who had a greater exposure to HIV care than the Jeddah sample. Most of the participants recruited from Jeddah were younger which means that they are at the beginning of their careers. These differences indicate the composition and should not be treated as characteristics related to a particular region.

Most of the HIV-related study variables were similar in both samples. However, some differences took place in case of Observed Stigma and Discrimination, and Health Facility Environment but these differences should be treated with caution due to the differences in samples and due to the exploratory nature of the bi-variate comparisons and the use of the cross-sectional approach.

The construct called earlier before as HIV knowledge was renamed as Fear of HIV Infection/Perceived Occupational Risk so as to be more precise about what was measured.

The multivariable model that assessed the influence of region and previous HIV training on attitudes toward PLHIV shows only limited results in terms of predicting attitudes toward PLHIV with only 0.6% of variance in attitude scores being explained. In general, the research demonstrates that HIV-related stigmatization is a multidimensional process and calls for further longitudinal and intervention studies in order to study the role in stigmatization of various personal, social, and organizational factors influencing HIV stigma in medical settings.

### Recommendations

In light of the study findings and the Saudi health care context, the recommendations provided are evidence-based and may be considered as follows:

#### Targeted stigma-reduction interventions

- Provide programs targeted to changing the attitudes and behaviors of health care providers (especially in facilities with higher stigma); e.g., Riyadh.
- Include experiential learning, reflective practice, and direct interaction with people living with HIV (PLHIV) to counter misinformation and develop empathy (17, 30).

#### Strengthening institutional policies and visibility

- Develop, distribute and enforce clear institutional policies related to HIV in all health care facilities; improvements in institutional HIV policy strength reduce observed stigma (35).
- Provide constructive monitoring and evaluation systems to ensure that policies are integrated into practice.

#### Improving environmental support and training

- Invest in facility readiness, mentorship and supportive working environments, especially in facilities with younger and less experienced health care providers (e.g., Jeddah).
- Sustain and grow HIV training programs, recognizing the importance of both technical knowledge and ethical care practices that are free from stigmatizing attitudes (18, 36).
- Curriculum Engagement of Early-Career Professionals and Students
- Bring HIV curriculum to undergraduate and graduate health profession programs to provide evidence-based knowledge, ethical attitudes, and stigma-free practices early in their training.

### Future research directions

Future research should further validate these instruments across diverse cultural, educational, and professional settings and compare them with established measures of HIV knowledge, stigma, and attitudes. Multi-center national and international studies involving healthcare professionals from different institutions, regions, and countries are needed to enhance generalizability and examine the influence of cultural, educational, organizational, and healthcare-system factors on HIV-related knowledge, attitudes, and stigma. Future investigations should also explore determinants such as access to education, exposure to health programs, institutional support, and socioeconomic status to better understand variations between urban and rural populations. Longitudinal studies are recommended to assess how these factors influence changes in knowledge, attitudes, stigma, and behaviors over time. Additionally, future research should incorporate more objective measures of clinical behavior, including direct observation, supervisor evaluations, simulation-based assessments, and standardized patient encounters, to better understand how knowledge and attitudes translate into practice. Qualitative and mixed-methods studies may further elucidate the cultural, organizational, and psychosocial factors underlying HIV-related stigma. Finally, research should evaluate organizational-level interventions, including workplace climate initiatives, leadership support, and workforce diversity strategies, to identify effective approaches for reducing stigma and improving patient care outcomes.

## Data Availability

The original contributions presented in the study are included in the article/supplementary material, further inquiries can be directed to the corresponding author/s.
